# A Rapid Late Enhancement MRI Protocol Improves Differentiation between Brain Tumor Recurrence and Treatment-Related Contrast Enhancement of Brain Parenchyma

**DOI:** 10.3390/cancers14225523

**Published:** 2022-11-10

**Authors:** Neda Satvat, Oliver Korczynski, Matthias Müller-Eschner, Ahmed E. Othman, Vanessa Schöffling, Naureen Keric, Florian Ringel, Clemens Sommer, Marc A. Brockmann, Sebastian Reder

**Affiliations:** 1 Department of Neuroradiology, University Medical Centre, Johannes Gutenberg-University of Mainz, 55131 Mainz, Germany; 2 Department of Neurosurgery, University Medical Centre, Johannes Gutenberg-University of Mainz, 55131 Mainz, Germany; 3 Department of Neuropathology, University Medical Centre, Johannes Gutenberg-University of Mainz, 55131 Mainz, Germany

**Keywords:** late enhancement, pseudo progression, radiation necrosis, glioblastoma, delayed-contrast-MRI

## Abstract

**Simple Summary:**

Differentiation between recurrence of malignant glioma and treatment-related changes (such as radiation necrosis) after radiochemotherapy using MRI can be challenging even for experienced neuroradiologists, as both entities may have a similar appearance regarding contrast enhancement and T2-signal. Using conventional MRI-sequences, early etiological assignment of a lesion can thus be difficult, although early and correct differentiation is mandatory in order to optimize individualized patient treatment. Late enhancement MRI up to 75 min after contrast administration has been described to improve differentiation between recurrent tumor tissue and treatment associated changes, but may complicate clinical workflow. We found that a more rapid late enhancement protocol still improves the specificity of follow-up MR imaging in patients with high-grade glioma, while reducing magnet time.

**Abstract:**

Purpose: Differentiation between tumor recurrence and treatment-related contrast enhancement in MRI can be difficult. Late enhancement MRI up to 75 min after contrast agent application has been shown to improve differentiation between tumor recurrence and treatment-related changes. We investigated the diagnostic performance of late enhancement using a rapid MRI protocol optimized for clinical workflow. Methods: Twenty-three patients with 28 lesions suspected for glioma recurrence underwent MRI including T1-MPRAGE-series acquired 2 and 20 min after contrast agent administration. Early contrast series were subtracted from late contrast series using motion correction. Contrast enhancing lesions were retrospectively and independently evaluated by two readers blinded to the patients’ later clinical course and histology with or without the use of late enhancement series. Sensitivity, specificity, NPV, and PPV were calculated for both readers by comparing results of MRI with histological samples. Results: Using standard MR sequences, sensitivity, specificity, PPV, and NPV were 0.84, 0, 0.875, and 0 (reader 1) and 0.92, 0, 0.885, and 0 (reader 2), respectively. Early late enhancement increased sensitivity, specificity, PPV, and NPV to 1 for each value and for both readers. Inter-reader reliability increased from 0.632 (standard MRI sequences) to 1.0 (with early late enhancement). Conclusion: The described rapid late enhancement MRI protocol improves MRI-based discrimination between tumor tissue and treatment-related changes of the brain parenchyma.

## 1. Introduction

With an average age-adjusted incidence rate of 3.2 per 100,000 [1,2,3], glioblastoma multiforme is the most common primary malignant brain tumor, accounting for about 16 percent of all primary brain neoplasms [4]. Although gross total resection of the contrast enhancing part of the tumor significantly improves survival times, it does not sufficiently prevent later recurrence or finally progression of the tumor [5,6,7]. Therefore, current standard therapy, in addition to surgical excision, includes radiation therapy (RT) with concurrent temozolomide (TMZ), an alkylating chemotherapeutic agent, followed by adjuvant chemotherapy with TMZ, also referred to as the “Stupp regimen” [8].

Radiochemotherapy (RCT) itself, however, in approximately 30% of patients results in contrast enhancing lesions mimicking tumor recurrence. One of these tumor mimics is radiation necrosis, observed several months and up to years after radiation therapy, is defined by delayed irreversible damage to blood vessels, leading to ischemic necrosis, demyelination, and hemorrhage [9]. Pseudo progression is a combination of regional inflammation-associated edema and increased vessel permeability without tumoral cell growth, usually frequently occurring within a few months after the end of radiation therapy [10,11]. As both processes show contrast enhancement within the treatment region, it can be difficult to distinguish therapy-related contrast enhancement of brain parenchyma from tumor recurrence or tumor progression. However, as the differentiation between tumor relapse and treatment-related contrast enhancement (TRCE) significantly affects a patient’s future treatment, the correct distinction between tumor recurrence and TRCE is of immanent importance.

To differentiate between tumor recurrence and TRCE more reliably, studies involving conventional MRI, MR spectroscopy, perfusion MR, and PET were conducted [10,12]. Although implementation of the aforementioned techniques and modalities improved diagnostic value, none of them provided ultimate assurance [7].

TREC and neoplastic tissue differ in their histopathological characteristics, and thus in their pathophysiological properties to take up and wash out contrast agent [10,12,13,14]. It has thus been investigated whether differences in contrast agent extravasation can be used to better discriminate between TRCE and tumor recurrence [15,16,17]. In these initial trials, high-resolution T1-weighted MR images were acquired up to 75 min after contrast injection and then subtracted to better visualize the “wash out” and “pooling” of contrast agent in suspicious regions. Contrast agent wash out has been reported to be an indicator of tumor recurrence with a sensitivity and positive predictive value (PPV) of 100% and 92%, respectively [15]. Despite these promising results, this “very late” enhancement measured 75 min after contrast agent administration meant a substantially longer waiting time for the patient and required additional magnet time. Inspired by the positive results of the above-mentioned studies, we implemented the methodology of late enhancement in our MRI protocol. We, however, modified the MR protocol in two ways: first, we cut down the time between the two post-contrast series to 20 min, hereby optimizing practicability of this protocol and, second, we used a commercially available, CE-certified software solution to perform subtraction of both post-contrast series in combination with motion correction.

The aim was to establish a simple and easy-to-use protocol for everyday clinical practice requiring less magnet time while improving decision making in neuro oncological imaging.

## 2. Materials and Methods

### 2.1. Patient Population

**Figure 1:**
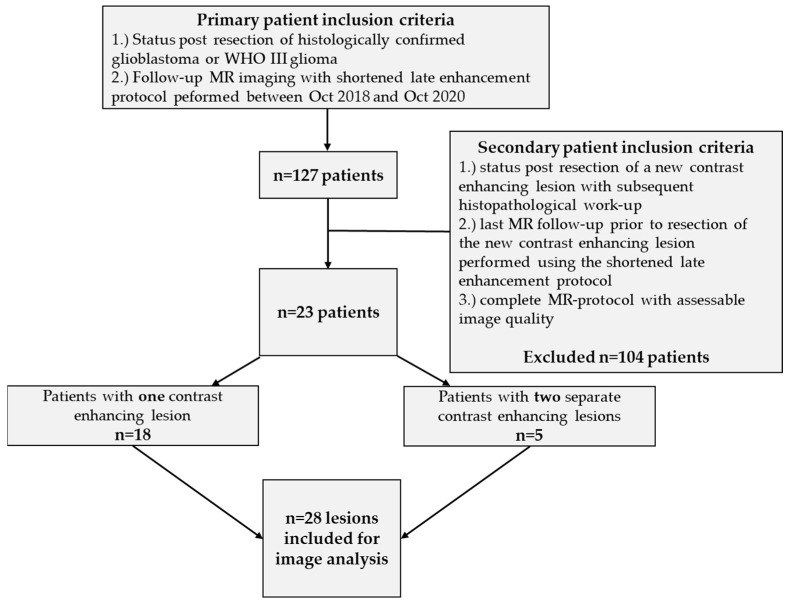
Patient acquisition and inclusion criteria.

We routinely implemented the MRI protocol described below so that all of our neuro oncological patients with high-grade primary brain tumors underwent the extended MRI protocol including late enhancement series. All patients were older than 18 years and previously underwent gross total tumor resection and RCT. However, as no experience regarding the interpretation of late enhancement series in our department existed, we decided to first collect a number of datasets between October 2018 and October 2020 in order to gain experience with late enhancement (late enhancement series were not considered for decision making during this period of time). This resulted in 127 rapid follow-up MRI protocols (including late enhancement) of patients with contrast enhancing brain tumors (GBM: *n* = 67 and WHO III glioma: *n* = 60). As shown in Figure 1, in this collective of 127 MRI scans, we retrospectively identified 23 patients who, after primary surgical therapy, completing RCT or RT, underwent another resection of tumor suspicious contrast enhancing lesions with subsequent histopathological workup of suspected recurrent glioma. This was regardless of the time interval between RCT and first MR follow-up with the MR protocol described below. In five patients, two histopathological findings were available for lesions at distant localizations, resulting in 28 cases eligible for subsequent analyses (see Appendix A, Table A1).

### 2.2. MRI-Protocol

All scans were acquired using a 1.5 T (Magnetom Sola, 20-channel head coil, Siemens Healthineers, Erlangen, Germany) or 3.0 T MR Scanner (Magnetom Skyra, 64-channel head coil, Siemens Healthineers).

**Figure 2:**
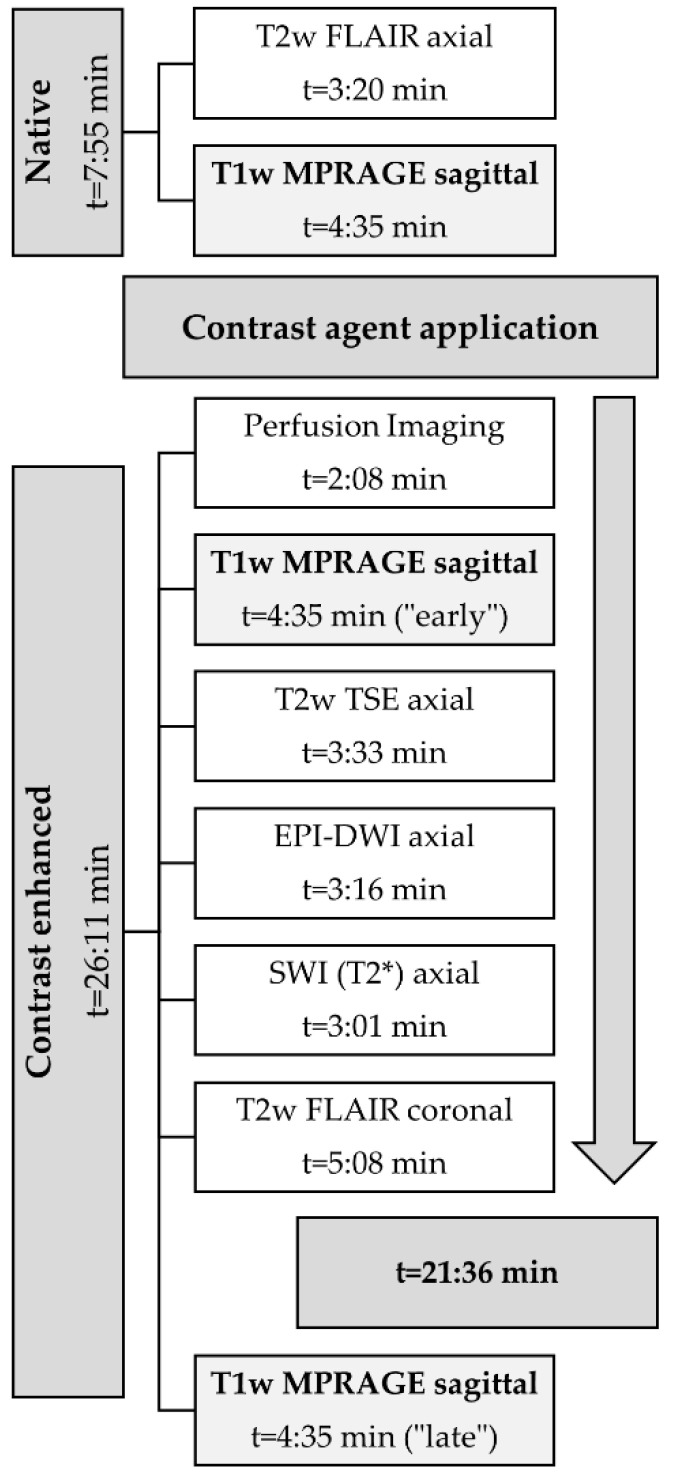
MR protocol.

The protocol comprised a non-enhanced T2 FLAIR and a T1-MPRAGE sequence. The 3D T1-MPRAGE sequences were acquired with TE/TR = 2.55/2200 ms, a field of view of 250 mm, a slice thickness of 1 mm, 192 slices, and an image matrix of 246 × 256. Next, a standard single dose of a Gadolinium-containing contrast agent (0.1 mmol/kg body weight; Gadovist^®^; Bayer Vital GmbH, Leverkusen, Germany) was injected intravenously using an automatic injection system. During the injection of the contrast agent, an MR perfusion sequence (DSC-MRI) was acquired. Next, the “early” (i.e., 2 min after contrast administration) T1-MPRAGE was acquired (sequence parameters as described above), followed by a T2 TSE, T2*, EPI-DWI, FLAIR, and the “late enhancement” T1-MPRAGE (i.e., twenty minutes after contrast administration) sequence, as shown in Figure 2. For evaluation of late enhancement, the 20′ post-contrast T1-MPRAGE was subtracted from the earlier 2′ post-contrast T1-MPRAGE after motion correction using the Syngo MR XA20 Software (Siemens Healthineers, Erlangen, Germany).

### 2.3. Evaluation of Late Enhancement

Retrospective analysis of MRI was performed by two experienced neuroradiologists. All MRI datasets were provided to the neuroradiologists in anonymized form.

As a first step, both neuroradiologists independently evaluated MRI data excluding the late enhancement series or subtraction maps regarding the question of whether the observed contrast enhancement was consistent with therapy-associated changes or tumor recurrence. Only datasets from patients with contrast enhancing lesions within initial MRI were included. The readers had access to all relevant clinical information, such as the initial histopathologically confirmed diagnosis, recent and current clinical status of the patient, as well as all treatment modalities including information on recent and current RCT until the time point of the underlying MRI. Image analyses were performed using, in our department, the routinely used image viewer (Sectra Medical Systems GmbH, Cologne, Germany).

**Figure 3:**
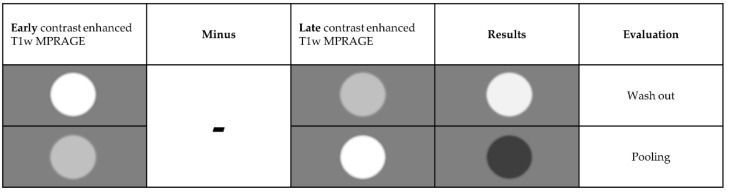
Principle of wash out and pooling. Tissue with higher contrast enhancement in early phase than in the late phase was labeled as **wash out** and has been evaluated as “suspect” (i.e., tumor recurrence). On the other hand, tissue with a continuous increase in contrast agent enhancement over time was labeled as **pooling** and evaluated as “not suspect” (TRCE; such as “pseudo tumors” or “pseudo progression”). Subtraction of late from early phase resulted in bright signals for suspect lesions and dark correlates for no suspect (see following figures).

**Figure 4:**
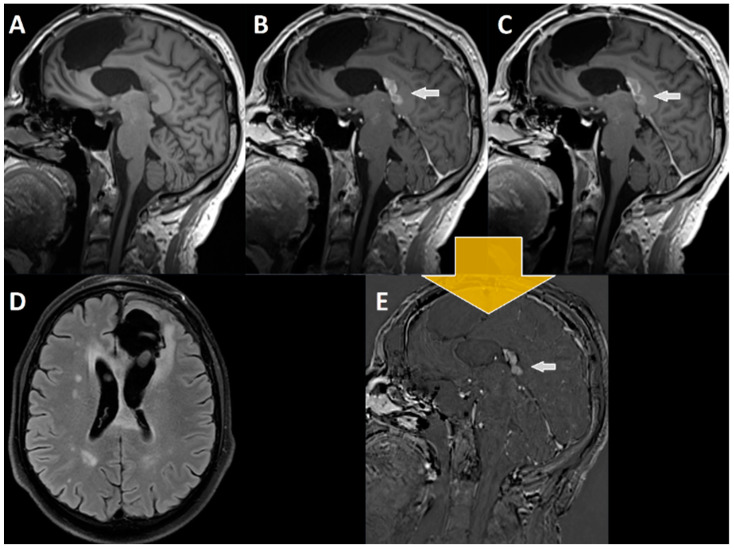
MRI imaging of a 52-year-old patient with glioblastoma, 7 months after resection and 4 months after RCT with discontinuation of adjuvant temozolomide therapy (**A**) T1-MPRAGE prior to i.v. administration of contrast agent, (**B**) early T1-MPRAGE 2 min after contrast administration, and (**C**) late T1-MPRAGE 20 min after contrast administration. (**D**) FLAIR sequence with the suspicious lesion in the corpus callosum. The white arrow in (**B**,**C**,**E**) points towards the contrast enhancing lesion suspicious of tumor recurrence. Subtraction of late (20 min, (**C**)) from early enhancement (2 min, (**B**)) T1-MPRAGE results (yellow arrow) in (**E**), where a **“wash out”** of contrast agent can be seen (**B** – **C** = **E**). Therefore, this lesion was retrospectively considered tumor tissue, which again was confirmed by postoperatively performed histopathological analyses.

**Figure 5:**
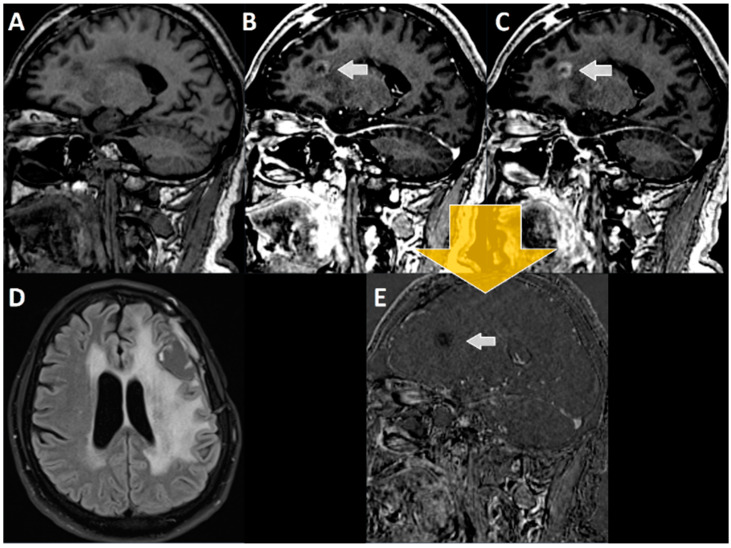
MRI imaging of a 66-year-old patient with glioblastoma, 21 months after resection and 18 months after combined radiochemotherapy with discontinuation of temozolomide therapy after the third cycle and 6 months after re-radiotherapy. (**A**) Non-enhanced, sagittal T1-MPRAGE; (**B**) early T1-MPRAGE 2 min after contrast administration; and (**C**) late T1-MPRAGE 20 min after contrast administration. The white arrow points towards the suspected left frontal lesion. (**D**) FLAIR imaging in transversal orientation. (**E**) The subtraction image (**B** – **C** = **E**). In the subtraction images, the lesion does not show any uptake, unlike the venous vessels (which typically, after 20 min, show a physiological wash out). The suspicious lesion thus shows a **“pooling”** of contrast agent and, therefore, is not suspicious for tumor recurrence, but for TRCE (which was confirmed in histopathological analyses).

Only after making a decision on the etiology of the contrast enhancing lesion(s) were the neuroradiologists allowed to reassess the MR images including the late enhancement and motion-corrected subtraction series. Tumor progression or recurrence was considered if a decrease in signal intensity from the early to the late enhancement T1-MPRAGE (i.e., 20 min after contrast agent) was observed (i.e., “wash out”). An increase in signal intensity from the early to the late enhancement series was indicative of therapy-induced changes or TRCE (i.e., “pooling” regarding pseudo progression, pseudo tumor, or radiation necrosis). Figure 3, Figure 4 and Figure 5 illustrate examples of contrast agent wash out (i.e., tumor recurrence/progression) and contrast agent pooling (i.e., therapy-associated changes) when using subtraction series.

### 2.4. Statistics

For statistical evaluation, the number of true positive/negative and false positive/negative diagnoses was determined and the sensitivity, specificity, PPV, and NPV were calculated. The results of the histopathological analyses of the resected tissue served as the gold standard. The values were calculated separately for both readers and for the cases without late enhancement and with late enhancement. In addition, an interrater reliability analysis was performed using Cohen’s kappa. All statistical analyses were performed using SPSS v 23.0 (IBM, Armonk, NY, USA).

## 3. Results

We retrospectively identified 28 lesions in 23 patients who underwent re-resection of suspected tumor tissue within a period of 24 months. Nine patients with ten lesions were female, the mean age of all patients was 58.3 ± 11.5 years, the mean follow-up time since the initial tumor resection was 10.4 ± 5.9 months, and the mean time between last MR follow-up and re-resection was 11.7 ± 9.6 days. After first resection, of these 23 patients, 20 patients were histologically diagnosed with GBM, 1 patient with diffuse astrocytic Glioma (WHO III), 1 with anaplastic astrocytoma (WHO III), and 1 patient with Gliosarcoma (WHO IV). The mean time between end of RT and of RCT and last MR follow-up before re-resection was 12 ± 10 months and 20 ± 21 months, respectively. The chemotherapeutic agent was temozolomide 75–200 mg/m^2^ and the mean dose of RT was 58.2 ± 9.1 Gy. Four patients received radiotherapy in addition to radiochemotherapy during follow-up. Two patients received re-challenge therapy with lomustine with 100 mg/m^2^. Three patients received temozolomide 75 mg/m^2^ at the time of MR follow-up prior to re-resection.

Consideration of late enhancement series resulted in an increase in sensitivity to detect tumor progress or recurrence from 84% for reader 1 (95% CI: 0.69–0.98) and 92.0% for reader 2 (95% CI: 0.82–1) to 100% (for both readers), respectively. Furthermore, the PPV increased from 87.5% (reader 1) and 88.0% (reader 2) to 100% (both readers). The specificity and NPV when including the late enhancement series for image interpretation were 100% for both readers. Detailed values are provided for each reader in Table 1.

The interrater reliability increased from substantial agreement (K = 0.632; *p* < 0.001) with both readers using only the normal MRI protocol with early enhancement series only to perfect agreement (K = 1.0; *p* < 0.001) when both readers additionally considered the subtracted and motion compensated late enhancement series.

## 4. Discussion

GBM is the most common as well as the most aggressive type of primary brain tumors in adults. Standard therapy for this tumor entity is surgical resection followed by radiochemotherapy. MR imaging is the standard method for monitoring disease progression and treatment response in patients suffering from high-grade glioma. Whereas contrast agent up-take within the treatment area is frequently observed in follow-up MRI, the underlying pathologies leading to contrast up-take are as different as they can be. The two most frequently encountered tissue alterations are therapy-related contrast enhancement (TRCE, in the case of radiation necrosis or pseudo progression) and, on the other hand, tumor-related contrast enhancement (i.e., tumor recurrence or tumor progression). Two therapy-associated changes were, as examples, radiation necrosis and pseudo progression. Most important is the differentiation between two lookalike lesions: (i) pseudo progression (therapy-associated) and (ii) tumor recurrence/progression. Between these conditions, a reliable distinction is crucial. In this context, treatment with intracavitary carmustin wafers has also been reported to result in therapy-associated lesions, which occur frequently within the first two months after implantation [18]. This finding has also been described by Ulmer et al., who reported new contrast enhancing lesions in the vicinity of the resection margin in 30% of cases within the first week and in 75% within the first month after implantation of carmustin wafers [19].

As conventional MR imaging is unable to provide reliable results, different approaches to better distinguish tumor tissue from therapy-associated changes have been applied. These approaches included (among multiple others) MR spectroscopy, diffusion-weighted imaging, dynamic susceptibility contrast-enhanced perfusion imaging, as well as dynamic contrast-enhanced MR imaging [20].

More recently, Zach et al. showed late enhancement MRI to improve differentiation between tumor tissue and therapy-associated tissue changes [15]. Briefly, enhancement subtraction maps calculated from high resolution T1-weighted MR sequences acquired up to 75 min after contrast administration were used to improve differentiation of tumor tissue from TRCE. By doing so, Zach et al. were able to improve differentiation of tumorous tissue from therapy-associated changes, as they observed the contrast agent to be “washed out” of the tumorous tissue in the late enhancement series, whereas treatment-associated changes showed a continuously increasing enhancement up to 75 min after contrast administration. Based on these findings, Wagner et al. analyzed the time-dependent changes in lesion morphology on T1w sequences for up to 55 min in brain metastases after stereotactic radiosurgery (SRS) and reported a characteristic and significant difference for malignant tissue and radiation-induced tissue damage after SRS [17].

Although the methodology of late enhancement MRI yielded promising results, major drawbacks of this method were that (i) late enhancement required significantly prolonged scan times or the patients had to be returned to the MR scanner to allow late enhancement series between 30 and 75 min post-contrast injection and (ii) a proprietary software was used to perform subtraction of the early and late enhancement series. The first point may result in longer examination or waiting times for the patients (some of them being in a bad condition); problems due to the MR scanner being occupied at the required time slot (owing to emergencies or unexpected prolongation of the preceding MR examination); and, maybe most importantly, a larger logistical effort, cost of magnet time, and workload for the staff. Considering these drawbacks, we wondered whether this very promising protocol may be successfully shortened while still allowing to perform late enhancement series within a single MRI examination. According to Zach et al., a significant clearance or accumulation of contrast agent compared with a baseline-scan acquired directly after contrast administration can be observed as early as after 15 min. We thus adapted the late enhancement protocol so that the total acquisition time in our protocol was 33 min.

We found the shortened MRI protocol to work well in daily clinical routine as no patient had to be taken out of the scanner and returned to the magnet later, hereby saving us at least several more minutes for this procedure (even in mobile patients). More time is being saved, as no repetitive planning of the examination (localizers etc.) is required and patient alignment usually remains stable. Finally, all patients well tolerated the slight extension of total scan time. Thus, this method is not only less stressful for the patients, but also easier to organize for the staff in terms of workflow, which, in times of staff shortage and emergency examination requests, is crucial. Furthermore, late enhancement subtraction maps with motion correction allowed more precise localization of tumor tissue owing to their higher spatial resolution compared with perfusion maps. This could be beneficial if suspected tissue is in proximity to eloquent brain regions. In addition, in the case of stereotactic biopsies, the high-resolution T1w-images provided by this method may help to prevent the acquisition of false negative specimens. In terms of sensitivity and PPV, the shortened protocol did not cause significant differences in the results reported by Zach. et al., who reported sensitivity and PPV of 100% and 92%, respectively [15].

Tomura et al. investigated several imaging modalities, such as (11-) C-methionine PET, FDG-PET MR permeability imaging, and ADC, in patients with brain metastases after stereotactic irradiation and reported PET with 11C-methionine (MET) to provide a high accuracy of 0.9 in discriminating between radiation necrosis and recurrent tumor, whereas the contrast enhancement ratio with 0.81 was rated second-best [21]. In this study, MET-PET was shown to be superior to DCS-MR imaging, ADC, and especially FDG-PET. MET-PET, however, is not widely available as the short half-life of 11C requires a cyclotron in close proximity to the hospital.

Galldicks et al. reported amino acid PET tracers to allow more accurate assessment of post-therapeutic brain lesions [22]. Glioblastoma studies using fluoro-ethyl tyrosine (FET)-PET achieved a diagnostic accuracy of at least 85%, differentiating between both “early” pseudo progression (within 12 weeks) and “late” pseudo progression (more than 12 weeks) after the end of therapy and true tumor progression. Therefore, if conventional MRI does not allow clear differentiation between tumor progression and therapy-associated changes (such as TRCE), PET may be helpful.

The relatively high costs and effort impede PET from being routinely used in follow-up of patients with malignant glioma.

Other studies reported sensitivity and specificity of MR spectroscopy (MRS) to be as high as 88–94% and 71–86%, respectively, when differentiating between radiation-induced necrosis and tumor recurrence [23,24]. Choline has been shown to be the most important marker for differentiating between these two entities. A high choline metabolism indicates tumor growth and, especially, the choline/creatin ratio has been reported to indicate tumor progression. A low choline metabolism on the other hand strongly suggested therapy-induced effects [23]. Despite its high diagnostic accuracy, MRS again is not frequently used for follow-up imaging in malignant glioma compared with other imaging techniques, such as MR perfusion [25]. The reasons for this may be the increased technical effort, increased measurement times, and lower spatial resolution.

Regarding radiomics, Jing et al. were able to differentiate early tumor recurrence from pseudo progression when analyzing 2632 radiomic features (e.g., shape features and intensity) in 118 patients. Their method reached a sensitivity and specificity between 64% and 86% and between 61% and 86%, respectively, in discrimination suspect from post-therapeutic lesions (or TRCE) [11]. Perfusion-weighted sequences, such as the DSC and DCE, are most widely used as a diagnostic tool for everyday clinical use [25]. One of the parameters determined from this is the relative cerebral blood volume (rCBV) [26]. Larsen et al. investigated the correlation of CBV in contrast-enhanced lesions with FDG-PET studies in their DCE study. The results showed that stable and regressing lesions had low CBV and no metabolic activity on FDG-PET, whereas tumor recurrence showed high CBV with high metabolic activity on FDG-PET [27]. The advantage of MR perfusion is the short scan time; as such, a fast sequence can easily be acquired during contrast administration. Compared with DSC, DCE requires a longer scanning and data processing time [26]. Thust et al. reported on DSC studies and meta-analyses that achieved high sensitivity (up to 90%) and specificity (up to 88%) [25]. Patel et al. also reported high accuracy of DSC and DCE in their meta-analysis, in which 13 of 28 studies examined pseudo progression, with pooled sensitivity and specificity of 90% and 88%, respectively, for DSC and 89% and 85%, respectively, for DCE 23 [26]. However, the disadvantage here is also the low spatial resolution due to the fast acquisition time and the susceptibility artefacts. There are several larger, population-based studies that support MR perfusion imaging as a basic diagnostic tool for identifying suspect lesions. Regarding DCE-MRI, Zhang et al. in their meta-analysis of 40 studies including 876 cases showed a pooled sensitivity and specificity of 0.83 each in differentiation between tumor recurrence and pseudo progression [28].

In contrast, the shortened late enhancement protocol as applied in the underlying study achieved a sensitivity and specificity of 100%, can be easily applied in a clinical setting, and can be easily evaluated, hereby supporting decision-making. Compared with Zach et al., we did not use an institutionally developed in-house solution to generate subtraction maps, nor did we require a long post-processing time to generate subtraction maps, whereas the late-enhancement subtraction images in our study were created using a CE-certified software.

The underlying study has some limitations. A shortcoming of our study is the somewhat small number of lesions analyzed (*n* = 28), which is smaller than the number of patients included by Zach and colleagues (*n* = 47), who likewise underwent re-resection or biopsy to serve as a gold standard. On the other hand, our results showed clearly that analysis of only 28 lesions was sufficient to demonstrate a significant increase in sensitivity (reader 1 84.5% and reader 2 92%, both increased to 100%) and specificity (reader 1 87.5% and reader 2 88%, both increased to 100%) using the rapid late enhancement protocol. Furthermore, to prove that the shortened late enhancement protocol is not inferior to that of Zach et al., a comparison of the response assessment with “very” late enhancement images 75 min after contrast administration would have been ideal. However, as our study was primarily concerned with correctly assigning enhancement patterns in an “early” late enhancement series, we did not use this method for the time being. In addition, we also wanted to avoid inconvenience for the patients and still were able to show a substantial improvement in sensitivity and PPV.

## 5. Conclusions

The rapid late enhancement MRI protocol increases sensitivity and specificity to discriminate recurring high grade glioma from therapy-associated changes of the brain parenchyma when being compared with a histopathological gold standard. The reduction in time between the two contrast series improves the clinical applicability of this MR protocol with a special focus on the reduction in magnet time and patient comfort. Given the encouraging results, consistent with previously reported, larger future studies with higher numbers of patients are warranted.

## Figures and Tables

**Table 1:** Chi-square analysis.

MRI *without* Late Enhancement	Histology Positive for Tumor Progression or Recurrence		Histology Negative for Tumor Progression or Recurrence	
tumor progress or recurrence rated in MRI	True positives (a)		False negatives (b)	
	21 (R1)	23 (R2)	4 (R1)	2 (R2)
tumor progress or recurrence rated in MRI	False positives (c)		True negatives (d)	
	3 (R1)	3 (R2)	0 (R1)	0 (R2)
Sensitivity $\frac{a}{a+b}$	84% (R1)		92% (R2)	
Specificity $\frac{d}{d+c}$	0% (R1)		0% (R2)	
PPV $\frac{a}{a+c}$	87.5% (R1)		88.5% (R2)	
NPV $\frac{d}{d+b}$	0% (R1)		0% (R2)	
Phi contingency coefficient (r_Phi_)	−0.141 (R1)		−0.096 (R2)	
RATZ-index	−0.167 (R1)		−0.12 (R2)	
MRI *with* late enhancement				
tumor progress or recurrence rated in MRI	True positives (a)		False negatives (b)	
	25 (R1)	25 (R2)	0 (R1)	0 (R2)
no tumor progression or recurrence in MRI	False positives (c)		True negatives (d)	
	0 (R1)	0 (R2)	3 (R1)	3 (R2)
Sensitivity $\frac{a}{a+b}$	100% (R1)		100% (R2)	
Specificity $\frac{d}{d+c}$	100% (R1)		100% (R2)	
PPV $\frac{a}{a+c}$	100% (R1)		100% (R2)	
NPV $\frac{d}{d+b}$	100% (R1)		100% (R2)	
Phi contingency coefficient (r_Phi_)	1 (R1)		1 (R2)	
RATZ-index	1 (R1)		1 (R2)	
**Odds ratio** for detecting suspect changes	1.1 (95% CI: 0.98–1.3 for R1 and R2)			

R1: reader 1. R2: reader 2.

## Data Availability

Data are available at the Department of Neuroradiology at the University Medical Mainz and can be requested from the director (M.A. Brockmann, MD, MSc). Each request should be based on a scientific hypothesis and reviewed by a (local) ethical committee. Any request must be made in writing. Data will be saved for ten years after publishing (according to GCP-guidelines).

## Appendix A

**Table A1:** Study population characteristics.

Lesion ID	Patient ID and Initial Tumor Histology	Patient Age at Initial Diagnosis [Years]	Localization of the Initial Tumor (Prior to First Resection)	Time (in Months) between the End of RCT and Detection of the First Suspect Contrast Enhancing Lesion in Follow-Up MRI	Localization of Suspect Lesion in Follow-Up MRI (with Wash Out in Late Enhancement)	Time (in Months) between the Detection of the Suspect Lesion and MRI Using the Late Enhancement Protocol	Time (in Months) between the Initial Tumor Resection and Re-Resection after Late Enhancement- Follow Up	Histology of Resected Lesions Declared as Suspect in Late Enhancement Follow-Up MRI
1	GBM-9	62	Precentral left	1	Craniorostral margin of the resection cavity	22	25	Residual/recurrent tumor tissue of glioblastoma and reactive changes
2	GBM-10	56	Temporobasal left	21	Temporopolar left	1	63	Residual/recurrent tumor tissue of glioblastoma
3	GBM-24	64	Temporoparietal left	Deceased during RCT	Craniomedial circumference of theresection cavity	0	5	Residual/recurrent tumor tissue of glioblastoma
4	GBM-32	57	Temporal left	3	Entire circumferential of the resection cavity	1	7	Residual/recurrent tumor tissue of glioblastoma
5	GBM-37	41	Precentral right	1	Frontal right	2	100	Residual/recurrent tumor tissue of glioblastoma
6	GBM-40 *	63	Temporal left	10	Anterolateral margin of resection cavity	0	13	Cell-rich anaplastic glial tumor with tumor tissue necrosis
7	GBM-40 *	63	Temporal left	10	Craniomedial of the resection cavity	5	13	Anaplastic glial tumor tissue compatible with residual/recurrent recurrent tumor tissue of known glioblastoma
8	GBM-45	55	Occipital right	5 (only radiotherapy)	Ventral circumferential resection margin	0	8	Anaplastic glial tumor with tumor tissue necrosis is compatible with residual/recurrence of glioblastoma
9	GBM-47 *	47	Occipital left	11	Parietotemporal left	0	14	Moderately cell-rich recurrence of known glioblastoma
10	GBM-47 *	47	Occipital left	20	Rostromedial and dorsomedial circumference of resection cavity	0	22	Cell-rich anaplastic astrocytic tumor. Residual/recurrent tumor of glioblastoma
11	GBM-49	79	Occipital left	2 (only radiotherapy)	Temporopolar left	0	3	Anaplastic glial tumor with tumor tissue necrosis, compatible with residual/recurrent tumor tissue glioblastoma
12	GBM-50	46	Corpus Callosum bilaterally	5	Truncus of the corpus callosum	1	10	Compatible with residual/recurrent tumor tissue of glioblastoma
13	GBM-54	63	Temporomesial left	13	Subcortical temporolateral left	0	16	Compatible with residual/recur-rent tumor tissue of glioblastoma
14	GBM-55	69	Parietal right	14	Pons	0	17	Compatible with residual/recurrent tumor tissue of glioblastoma
15	GBM-56	23	Frontopolar right	31 (only radiotherapy)	Parietal right	0	36	Compatible with residual/recurrent tumor tissue of glioblastoma
16	GBM-57 *	57	Temporal left	3	Temporobasal left	0	6	Cell-rich anaplastic astrocytic tumor compatible with residual/recurrent tumor tissue of glioblastoma
17	GBM-57 *	57	Temporal left	14	Dorsal to the resection cavity around the left cornu posterior	0	17	Cell-rich anaplastic astrocytic tumor with high proliferation activity
18	GBM-49	52	Frontal right	27	Lateral to the right anterior cornu	0	33	Compatible with glioblastoma
19	GBM-60	70	Parietooccipital right	1	Anteromedial to the resection cavity	0	5	Glial tissue with increased cell density and reactive changes
20	GBM-63	43	Frontal left	not assessable, external image data	Posteromedial of the genu corporis callosi	not assessable, external image data	24	Compatible with residual/recurrent tumor tissue of glioblastoma
21	GBM-64	71	Frontal right	3	Resection margin	5	10	Compatible with residual/recurrent tumor tissue of glioblastoma
22	GBM-65	54	Frontal left	3	Frontal left margin of resection cavity	6	12	Cell-dense portions of a glial tumor, as well as reactive/resorptive changes
23	GBM-66	43	Temporal left	4	Resection margin	0	20	Residual/recurrent tumor tissue of glioblastoma
24	WHO3-11	62	Temporal left	5	Temporal left	0	8	Coagulation necrosis as a radiation regressive change. No malignant cells of the known anaplastic astrocytoma
25	WHO3-26 *	46	Temporal right	37	Parietal right/temporo-occipital right	0	40	Residual/recurrent tumor tissue of the known anaplastic astrocytoma
26	WHO3-26 *	46	Temporal right	47	In the medial temporal lobe right	0	49	Residual/recurrent tumor tissue of anaplastic astrocytoma
27	WHO3-53 *	62	Frontal right	0	Ventromedial circumference of resection cavity	9	13	Necrotic tissue particles and connective tissue with reactive/resorptive changes
28	WHO3-53 *	62	Frontal right	0	Ventrocaudal circumference of resection cavity	1	17	Extensive therapy-induced reactive and resorptive changes

* Two separate lesions in the same patient.
